# Bayesian sample size determination using commensurate priors to leverage preexperimental data

**DOI:** 10.1111/biom.13649

**Published:** 2022-03-28

**Authors:** Haiyan Zheng, Thomas Jaki, James M.S. Wason

**Affiliations:** ^1^ MRC Biostatistics Unit University of Cambridge Cambridge UK; ^2^ Population Health Sciences Institute Newcastle University Newcastle upon Tyne UK; ^3^ Department of Mathematics and Statistics Lancaster University Lancaster UK

**Keywords:** Bayesian experimental designs, historical data, rare‐disease trials, robustness, sample size

## Abstract

This paper develops Bayesian sample size formulae for experiments comparing two groups, where relevant preexperimental information from multiple sources can be incorporated in a robust prior to support both the design and analysis. We use commensurate predictive priors for borrowing of information and further place Gamma mixture priors on the precisions to account for preliminary belief about the pairwise (in)commensurability between parameters that underpin the historical and new experiments. Averaged over the probability space of the new experimental data, appropriate sample sizes are found according to criteria that control certain aspects of the posterior distribution, such as the coverage probability or length of a defined density region. Our Bayesian methodology can be applied to circumstances that compare two normal means, proportions, or event times. When nuisance parameters (such as variance) in the new experiment are unknown, a prior distribution can further be specified based on preexperimental data. Exact solutions are available based on most of the criteria considered for Bayesian sample size determination, while a search procedure is described in cases for which there are no closed‐form expressions. We illustrate the application of our sample size formulae in the design of clinical trials, where pretrial information is available to be leveraged. Hypothetical data examples, motivated by a rare‐disease trial with an elicited expert prior opinion, and a comprehensive performance evaluation of the proposed methodology are presented.

## 1 INTRODUCTION

Conventionally, sample size has often been determined to control certain aspects of the sampling distribution of a test statistic (Desu and Raghavarao, 1990). This is typically considered from a frequentist perspective that operating characteristics, for example, type I error rate and power, should be maintained for detecting a meaningful magnitude of the difference. For data that are assumed to be independently and identically distributed normal, sample size may be a function also of nuisance parameters such as unknown variances. Fixing such parameters to certain values may leave the determination inaccurate, or only locally optimal, as an arbitrary guess could deviate far from the true value. The Bayesian framework has been argued to be more advantageous to sample size determination (SSD), since it allows uncertainty to be described in a prior for the parameters (O'Hagan and Forster, 2004). Moreover, it brings about the possibility of incorporating preexperimental information, if available, in a prior for the parameter of interest and/or nuisance parameters. Considerable attention has thus been given to Bayesian SSD; see, for example, Clarke and Yuan (2006).

Two main kinds of methodology written in the literature are “hybrid classical and Bayesian” and “proper Bayesian” SSD (Spiegelhalter *et al.*, 2004). With the former, sample size would be chosen to ensure that the predictive power, obtained by averaging the frequentist power function over a prior distribution for the unknown parameter(s), reaches a desired target level. By contrast, “proper Bayesian” SSD approaches refer to those for which the final analysis of data would also be Bayesian. Joseph *et al.* (1995) derive formulae for binomial experiments comparing two proportions; specifically, sample sizes are sought to ensure, for example, an adequate coverage probability or width of a defined interval of the posterior “success” rate. Joseph and Bélisle (1997) concentrate on normal distributions and use normal‐gamma conjugate priors for experiments that estimate either single normal means or the difference between two normal means. In the context of clinical trials, Whitehead *et al.* (2008) develop Bayesian methods resembling frequentist formulations of the SSD problem in exploratory trials, to demonstrate the treatment effect based on posterior interval probabilities. These fully Bayesian approaches shed light on the option of incorporating preexperimental data into a prior for both the design and analysis consistently.

This paper is focused on fully Bayesian SSD permitting the use of preexperimental data from multiple sources. Our research is partly motivated by the efficient design and analysis of clinical trials that evaluate a new treatment for rare diseases (EMA, 2006), where asking for a sample size to achieve the frequentist power is often infeasible. Pretrial information, collected from historical studies which had been conducted under similar circumstances, or elicited from expert opinion, could play an essential role. The proposed methodology would nonetheless be generic: it can be applied to areas where there is a need to use preexperimental data formally through the mechanism of specifying priors. For instance, the sample size for environmental water quality evaluation could be limited: borrowing strength from historical water monitoring data has been considered helpful (Duan *et al.*, 2006).

## 2 METHODS

### 2.1 Borrowing of historical information from multiple sources

Suppose there are *K* relevant sets of data, $y_{1},\cdots ,y_{K}$, to specify a prior for the parameter, denoted by $\mu_{\Delta }$, underpinning a new experiment. Let $\theta_{1},\cdots ,\theta_{K}$ denote the counterparts of $\mu_{\Delta }$, specific to each historical experiment k=1,⋯,K. Following Zheng and Wason (2022), we specify *K* commensurate predictive distributions by the source of information, which are formulated as conditional normal distributions with an unknown mean $\theta_{k}$ and precision $\nu_{k}$ (the variance would thus be $\nu_{k}^{-1}$):

(1) $${\tilde{\theta }}_{k}|\theta_{k},\nu_{k}∼N\left(\theta_{k},\nu_{k}^{-1}\right),\;\text{for}\;k=1,\cdots ,K,$$

where each ${\tilde{\theta }}_{k}$ is regarded as *equivalent* to $\mu_{\Delta }$ in terms of the parameter space. More precisely, it means that the parameter space for ${\tilde{\theta }}_{k}$, as projected from a preexperimental parameter $\theta_{k}$, would be defined with the same or comparable set of parameter values to that of $\mu_{\Delta }$. The precision $\nu_{k}$ is sometimes referred to as a commensurate parameter. Different from the original proposal with a spike‐and‐slab prior on each $\nu_{k}$, we consider a mixture of conjugate priors for analytical derivations; that is, for the predictive precision:

(2) $$\begin{array}{c} \nu_{k}∼w_{k}\text{Gamma}\left(a_{01},b_{01}\right)+\left(1-w_{k}\right)\text{Gamma}\left(a_{02},b_{02}\right), \end{array}$$

where $w_{k}$ is the prior mixture weight, on the scale of [0, 1], to represent preliminary skepticism about how commensurate $\theta_{k}$ and $\mu_{\Delta }$ would be. The hyperparameters are chosen so that the first mixture component with $a_{01},b_{01}$ has the density concentrated on small values of $\nu_{k}$, while the second mixture component with $a_{02},b_{02}$ has density covering larger values of $\nu_{k}$. A large prior mixture weight allocated to either component distribution would thus result in sufficient down‐weighting (with no borrowing at all as one extreme) or strong borrowing of historical information (with fully pooling as the other extreme), respectively. Stipulating $0<w_{k}<1$ in (2) produces a compromise between the two extreme cases. The strength of this Gamma mixture prior is then tuned by $w_{k}$, which can be interpreted as the prior probability of incommensurability.

Then, $f({\tilde{\theta }}_{k},\nu_{k}|\theta_{k})$ has a Normal‐Gamma mixture distribution. By integrating out the nuisance parameter $\nu_{k}$, we further obtain

(3) $$\begin{array}{ccc} & & f\left({\tilde{\theta }}_{k}|\theta_{k}\right)\propto w_{k}{\left(\frac{{\left({\tilde{\theta }}_{k}-\theta_{k}\right)}^{2}}{2b_{01}}+1\right)}^{-\frac{2a_{01}+1}{2}}\\ & & \;+\left(1-w_{k}\right){\left(\frac{{\left({\tilde{\theta }}_{k}-\theta_{k}\right)}^{2}}{2b_{02}}+1\right)}^{-\frac{2a_{02}+1}{2}}, \end{array}$$

which is a two‐component mixture of nonstandardized (shifted and scaled) *t* distributions. In particular, the component *t* distributions have their location parameters identically as $\theta_{k}$ yet scale parameters as $\frac{b_{01}}{a_{01}}$ and $\frac{b_{02}}{a_{02}}$, respectively. Detailed derivation of (3) and the demonstration of it being a nonstandardized *t* mixture distribution are given in Section A of the Supporting Information. For easing the synthesis of *K* predictive priors later on, we approximate this unimodal *t* mixture distribution by a normal distribution that

(4) $$\begin{array}{ccc} & & {\tilde{\theta }}_{k}|\theta_{k}\;\dot{∼}\;N\left(\theta_{k},\frac{w_{k}b_{01}}{a_{01}-1}+\frac{\left(1-w_{k}\right)b_{02}}{a_{02}-1}\right),\\ & & \;\;\text{with}\;a_{01},a_{02}>1. \end{array}$$

This approximation is based on the first two moments of the nonstandardized *t* mixture distribution, which are analytically available; see Section B of the Supporting Information for details. The variance of the normal approximation takes account of the dispersion of both *t* mixture components. The goodness of such normal approximation to the original *t* mixture distribution depends on the degrees of freedom, 2*a*_01_ and 2*a*_02_, and the scale parameters, $\frac{b_{01}}{a_{01}}$ and $\frac{b_{02}}{a_{02}}$, which are of the investigators' choice. We show the numerical accuracy of this approximation in Section C of the Supporting Information.

With the normal approximation given by (4), we stipulate $\mu_{\Delta }$ as a linear combination of K≥2 hypothetical random variables, ${\tilde{\theta }}_{k}$, projected from the preexperimental parameters. That is, $\mu_{\Delta }=\sum_{k}p_{k}{\tilde{\theta }}_{k}$, for k=1,⋯,K. These synthesis weights $p_{1},\cdots ,p_{K}$ sum to 1, with each reflecting the relative importance of a corresponding preexperimental dataset to constitute the collective predictive prior for $\mu_{\Delta }$. Pragmatically, one may associate these synthesis weights with the prior probabilities of commensurability, that is, $1-w_{k}$, so that a preexperimental dataset thought of as more commensurate would be assigned a larger $p_{k}$ to derive the collective prior for $\mu_{\Delta }$. Applying the convolution operator for the sum of normal random variables, $\mu_{\Delta }$ has a normal prior distribution. Suppose each preexperimental dataset leads to an estimate of $\theta_{k}|y_{k}∼N\left(m_{k},s_{k}^{2}\right),\;k=1,\cdots ,K$. We thus obtain a normal collective prior that

(5) $$\mu_{\Delta }|y_{1},\cdots ,y_{K}\dot{∼}N\left(\sum_{k}p_{k}\lambda_{k},\sum_{k}p_{k}^{2}\xi_{k}^{2}\right),$$

with

(6) $$\begin{array}{ccc} & & \lambda_{k}=m_{k}\;\;\text{and}\;\;\xi_{k}^{2}=s_{k}^{2}+\frac{w_{k}b_{01}}{a_{01}-1}+\frac{\left(1-w_{k}\right)b_{02}}{a_{02}-1},\\ & & \;\;\;\;\;\;\;(a_{01},a_{02}>1) \end{array}$$

being the marginal prior means and variances. It accounts for both the variability in a preexperimental dataset ***y***_*k*_ and the postulated level of incommensurability, $w_{k}$, through the Gamma mixture prior placed on the predictive precision, $\nu_{k}$. We give more details in Section D of the Supporting Information for this derivation. Using Bayes' theorem, this collective prior will be updated by the new experimental data, denoted by $y_{K+1}$, to a robust posterior.

### 2.2 Criteria for the Bayesian SSD

Most Bayesian SSD criteria aim to control certain property of the posterior, denoted by $f_{p}\left(\mu_{\Delta }|y_{1},\cdots ,y_{K},y_{K+1}\right)$, wherein $y_{K+1}$ are unobserved at the design stage. It is important to state that uncertainty of sampling a set of data as $y_{K+1}$ from the entire *probability space* needs to be accounted for. Thus, strictly speaking, the Bayesian SSD criteria can only maintain the average properties of the posterior.

Joseph and Bélisle (1997) propose specifying a density region, $R(y_{K+1})$, bounded by *r* and $r+\ell_{0}$, to contain possible parameter values. Here, ℓ_0_ is the desired interval length and *r* chosen so that $R(y_{K+1})$ is the highest posterior density (HPD) interval; the so‐called HPD because this interval includes the mode of the posterior distribution. This specification can ensure the coverage probability of $R(y_{K+1})$ to be at least 1−α, when averaged over all possible samples. Formally, it requires that

(7) $$\begin{array}{c} \int_{Y}\left\{\int_{r}^{r+\ell_{0}}f_{p}\left(\mu_{\Delta }|y_{1},\cdots ,y_{K},y_{K+1}\right)d\mu_{\Delta }\right\}f_{d}\left(y_{K+1}\right)dy_{K+1}\geq 1-\alpha , \end{array}$$

where Y denotes the probability space and $f_{d}\left(y_{K+1}\right)$ the marginal distribution of the sample, that is, the new experimental data. For controlling the coverage probability, it is often referred to as the average coverage criterion (ACC). The posterior distribution in our context would be unimodal and symmetric about the posterior mean, as we can envisage from the collective prior given by (5). We would then simply stipulate the HPD interval as

(8) $$R\left(y_{K+1}\right)=E\left(\mu_{\Delta }|y_{1},\cdots ,y_{K},y_{K+1}\right)\pm \frac{\ell_{0}}{2},$$

which coincides with the alpha‐expectation tolerance region by Fraser and Guttman (1956).

An alternative to the ACC is the average length criterion (ALC), which limits the interval length to be at most ℓ for a posterior interval that has a coverage probability of $1-\alpha_{0}$ (Joseph and Bélisle, 1997). Let $\ell^{\prime }\left(y_{K+1}\right)$ be the random interval length of the posterior credible interval dependent on the unobserved new experimental data. Targeting a fixed coverage probability of $1-\alpha_{0}$, one may solve $\ell^{\prime }\left(y_{K+1}\right)$ to meet

(9) $$\int_{r}^{r+\ell^{\prime }\left(y_{K+1}\right)}f_{p}\left(\mu_{\Delta }|y_{1},\cdots ,y_{K},y_{K+1}\right)d\mu_{\Delta }=1-\alpha_{0},$$

where *r* would be specified to give the HPD interval as that for the ACC above. Averaged over all possible samples, the ALC requires that

(10) $$\int_{Y}\ell^{\prime }\left(y_{K+1}\right)f_{d}\left(y_{K+1}\right)dy_{K+1}\leq \ell .$$

The ALC could be more favored than the ACC, since Bayesian practitioners are keen to report, for example, a 95% credible interval for the posterior mean, in the analysis.

As we can see from (7) and (10), sample sizes chosen to meet the ACC or ALC rely on the marginal, predictive distribution of $y_{K+1}$; that is, $f_{d}\left(y_{K+1}\right)=\int f\left(y_{K+1}|\mu_{\Delta }\right)\pi \left(\mu_{\Delta }\right)d\mu_{\Delta }$. When $f_{d}\left(y_{K+1}\right)$ also depends on nuisance parameters, say the variance $\sigma_{0}^{2}$ being unknown, it becomes $f_{d}\left(y_{K+1}\right)=\int \int f\left(y_{K+1}|\mu_{\Delta },\sigma_{0}^{2}\right)\pi \left(\mu_{\Delta }\right)g\left(\sigma_{0}^{2}\right)d\mu_{\Delta }d\sigma_{0}^{2}$. In our context, priors for unknown $\mu_{\Delta }$ and $\sigma_{0}^{2}$ would be specified based on preexperimental information. The predictive distribution $f(y_{K+1})$ would thus formally be $f_{d}\left(y_{K+1}|y_{1},\cdots ,y_{K}\right)$, given our $\pi (\mu_{\Delta }|y_{1},\cdots ,y_{K})$ and $g(\sigma_{0}^{2}|y_{1},\cdots ,y_{K})$.

We consider one additional criterion relating to the moments of posterior distribution. For practical reasons, we focus on the second central moment only, so the criterion would be referred to as the average posterior variance criterion (APVC) hereafter. Given a fixed level of dispersion ε_0_, a suitable sample size is chosen to ensure that

(11) $$E_{Y}\left[\text{Var}\left(\mu_{\Delta }|y_{1},\cdots ,y_{K},y_{K+1}\right)\right]\leq \varepsilon_{0}.$$

As Adcock (1997) commented, this criterion is equivalent to using the *L*_2_‐norm loss function for inferences: $L_{2}\left(\mu_{\Delta }\right)={\left(\mu_{\Delta }-E\left(\mu_{\Delta }|y_{1},\cdots ,y_{K},y_{K+1}\right)\right)}^{2}$. It is also worth noting that the literature also documented many other Bayesian approaches to SSD, for example, based on the use of utility theory (Lindley, 1997) and Bayes factors (Weiss, 1997); the latter is relevant to pursuing the control of type I error rate and power in hypothesis testing problems.

We note that the fixed values of ℓ_0_, α_0_, and ε_0_ are all positive real numbers. Unlike the frequentist statistical significance levels, there is no convention to set these thresholds. It is most likely to be backed up by supporting details from the field of application; questions such as what is the meaningful range of $\mu_{\Delta }$ that can provide compelling evidence for the inference may be discussed with a subject‐matter expert.

### 2.3 Sample size required for comparing two normal means

Consider the comparison of two normal means, denoted by $\mu_{j},\;j=A,B$, in a new experiment. The difference $\mu_{\Delta }=\mu_{A}-\mu_{B}>0$ would indicate *A* is superior to *B*. Let $X_{ij}$ be the measured outcome from experimental unit *i* assigned to group *j*. We assume these measurements are independent, random samples drawn from populations with overall mean $\mu_{j}$ and a common variance $\sigma_{0}^{2}$. Letting $n_{j}$ be the groupwise sample sizes, the sample means ${\bar{X}}_{j}=\left(X_{1j}+\cdots +X_{n_{j}j}\right)/n_{j}$ follow an asymptotic normal distribution by the central limit theorem; that is, ${\bar{X}}_{j}∼N\left(\mu_{j},\frac{\sigma_{0}^{2}}{n_{j}}\right)$, for j=A,B. This further leads to ${\bar{X}}_{\Delta }∼N\left(\mu_{\Delta },\frac{\sigma_{0}^{2}}{n_{A}}+\frac{\sigma_{0}^{2}}{n_{B}}\right)$.

#### 2.3.1 For cases of known variance

When the common variance $\sigma_{0}^{2}$ is known, $\mu_{\Delta }=\mu_{A}-\mu_{B}$ has a normal prior based on preexperimental datasets $y_{1},\cdots ,y_{K}$, as was given in (5). Since the joint likelihood of the $n_{A}+n_{B}$ measurements in the new experiment $L\left(y_{K+1}|\mu_{A},\mu_{B}\right)\propto L\left({\bar{X}}_{\Delta }|\mu_{A},\mu_{B}\right)$, we formulate the data likelihood in terms of ${\bar{X}}_{\Delta }$, which is regarded as a random variable. We further derive the posterior distribution as

(12) $$\begin{array}{c} \mu_{\Delta }|y_{1},\cdots ,y_{K},y_{K+1}\dot{∼}N\left(\eta ,{\left(\frac{1}{\sum p_{k}^{2}\xi_{k}^{2}}+\frac{1}{\left(\frac{1}{n_{A}}+\frac{1}{n_{B}}\right)\sigma_{0}^{2}}\right)}^{-1}\right), \end{array}$$

with

(13) $$\begin{array}{ccc} & & \eta =\frac{\left(\frac{1}{n_{A}}+\frac{1}{n_{B}}\right)\sigma_{0}^{2}}{\sum p_{k}^{2}\xi_{k}^{2}+\left(\frac{1}{n_{A}}+\frac{1}{n_{B}}\right)\sigma_{0}^{2}}\sum p_{k}\lambda_{k}\\ & & \;+\frac{\sum p_{k}^{2}\xi_{k}^{2}}{\sum p_{k}^{2}\xi_{k}^{2}+\left(\frac{1}{n_{A}}+\frac{1}{n_{B}}\right)\sigma_{0}^{2}}{\bar{x}}_{\Delta }, \end{array}$$

where ${\bar{x}}_{\Delta }$ is the realization of ${\bar{X}}_{\Delta }$. The marginal distribution (unconditional on $\mu_{\Delta }$) for the difference in sample means is

(14) $$\begin{array}{c} {\bar{X}}_{\Delta }|y_{1},\cdots ,y_{K}\dot{∼}N\left(\sum_{k}p_{k}\lambda_{k},\left(\frac{1}{n_{A}}+\frac{1}{n_{B}}\right)\sigma_{0}^{2}+\sum_{k}p_{k}^{2}\xi_{k}^{2}\right), \end{array}$$

which corresponds to $f_{d}\left(y_{K+1}\right)$ in Section 2.2.

As Joseph and Bélisle (1997) noted, the ACC and ALC result in the same outcome for cases where the variance is known. Hence, we illustrate using the ACC in the following. Letting the HPD interval $\left(r,r+\ell_{0}\right)$ stretch symmetrically around the posterior mean η, the coverage can be computed by

(15) $$\begin{array}{ccc} & & P\left[|\mu_{\Delta }-\eta |\leq \frac{\ell_{0}}{2}|y_{1},\cdots ,y_{K},y_{K+1}\right]\\ & & \;=\Phi \left(\sqrt{\frac{1}{\sum p_{k}^{2}\xi_{k}^{2}}+\frac{1}{\left(\frac{1}{n_{A}}+\frac{1}{n_{B}}\right)\sigma_{0}^{2}}}\frac{\ell_{0}}{2}\right), \end{array}$$

where Φ(·) is the cumulative distribution function of the standard normal distribution. We thus have $\Phi (\sqrt{\frac{1}{\sum p_{k}^{2}\xi_{k}^{2}}+\frac{1}{\left(\frac{1}{n_{A}}+\frac{1}{n_{B}}\right)\sigma_{0}^{2}}}\frac{\ell_{0}}{2})\geq 1-\alpha$, which is rearranged as

(16) $$\frac{n_{A}n_{B}}{n_{A}+n_{B}}\geq \left(\frac{4z_{\alpha /2}^{2}}{\ell_{0}^{2}}-\frac{1}{\sum p_{k}^{2}\xi_{k}^{2}}\right)\sigma_{0}^{2},$$

where $z_{\alpha /2}$ is the upper (α/2)‐th quantile of the standard normal distribution, that is, $\Phi^{-1}\left(1-\alpha /2\right)$. Similarly, averaging over the entire data space, the APVC gives

(17) $$\frac{n_{A}n_{B}}{n_{A}+n_{B}}\geq \left(\frac{1}{\varepsilon_{0}}-\frac{1}{\sum p_{k}^{2}\xi_{k}^{2}}\right)\sigma_{0}^{2}.$$

When $\sum p_{k}^{2}\xi_{k}^{2}$, the prior variance for $\mu_{\Delta }$ based on preexperimental data is so small that the right‐hand side of the inequalities above becomes zero or negative, little or no information would be required to accrue from a new experiment.

#### 2.3.2 For cases of unknown variance

When $\sigma_{0}^{2}$ is unknown, we assume that the quantity $c\sum p_{k}^{2}\xi_{k}^{2}/\sigma_{0}^{2}∼\chi^{2}\left(c\right)$, where $\chi^{2}\left(c\right)$ refers to a chi‐square distribution with *c* degrees of freedom (Gelman *et al.*, 2013). This is equivalent to specifying that $\sigma_{0}^{2}∼$ Inv‐Gamma($\frac{c}{2},\frac{c\sum p_{k}^{2}\xi_{k}^{2}}{2}$); hence, the larger value *c* takes, the more $\sigma_{0}^{2}$ converges to the prior variance for $\mu_{\Delta }|y_{1},\cdots ,y_{K}$. The marginal posterior for $\mu_{\Delta }$ will then be obtained by integrating out the nuisance parameter $\sigma_{0}^{2}$:

(18) $$\begin{array}{ccc} & & f_{p}\left(\mu_{\Delta }|y_{1},\cdots ,y_{K},y_{K+1}\right)\\ & & \;=\int \pi_{p}\left(\mu_{\Delta },\sigma_{0}^{2}|y_{1},\cdots ,y_{K},y_{K+1}\right)g\left(\sigma_{0}^{2}\right)d\sigma_{0}^{2}\\ & & \;\propto \exp\left(-\frac{{\left(\mu_{\Delta }-\sum p_{k}\lambda_{k}\right)}^{2}}{2\sum p_{k}^{2}\xi_{k}^{2}}\right){\left[1+\frac{1}{c}\cdot \frac{{\left(\mu_{\Delta }-{\bar{x}}_{\Delta }\right)}^{2}}{\left(\frac{1}{n_{A}}+\frac{1}{n_{B}}\right)\sum p_{k}^{2}\xi_{k}^{2}}\right]}^{-\frac{c+1}{2}}, \end{array}$$

that is, the posterior is proportional to the product of normal and nonstandardized *t* kernels (Ahsanullah *et al.*, 2014). Detailed steps for deriving (18) are given in Section E of the Supporting Information. In particular, the *t* density kernel (with the location and scale parameters being ${\bar{x}}_{\Delta }$ and $\left(\frac{1}{n_{A}}+\frac{1}{n_{B}}\right)\sum p_{k}^{2}\xi_{k}^{2}$, respectively) can be related to a normal kernel with the same location parameter and the variance as $\left(\frac{1}{n_{A}}+\frac{1}{n_{B}}\right)\sigma_{0}^{2}$, conditional on $c\sum p_{k}^{2}\xi_{k}^{2}/\sigma_{0}^{2}∼\chi^{2}\left(c\right)$. The posterior (18) can thus be further developed as

(19) $$\begin{array}{ccc} & & f_{p}\left(\mu_{\Delta }|\sigma_{0}^{2},y_{1},\cdots ,y_{K},y_{K+1}\right)\\ & & \;\propto \exp\left(-\frac{{\left(\mu_{\Delta }-\sum p_{k}\lambda_{k}\right)}^{2}}{2\sum p_{k}^{2}\xi_{k}^{2}}\right)\exp\left(-\frac{{\left(\mu_{\Delta }-{\bar{x}}_{\Delta }\right)}^{2}}{2\left(\frac{1}{n_{A}}+\frac{1}{n_{B}}\right)\sigma_{0}^{2}}\right)\\ & & \;\overset{\mathrm{def}}{=}\exp\left(-\frac{{\left(\mu_{\Delta }-\mu_{N}\right)}^{2}}{2\sigma_{N}^{2}}\right), \end{array}$$

with

(20) $$\sigma_{N}^{2}={\left(\frac{1}{\sum p_{k}^{2}\xi_{k}^{2}}+\frac{1}{\left(\frac{1}{n_{A}}+\frac{1}{n_{B}}\right)\sigma_{0}^{2}}\right)}^{-1},$$

which is consistent with (12) but here with unknown $\sigma_{0}^{2}∼$ Inv‐Gamma($\frac{c}{2},\frac{c\sum p_{k}^{2}\xi_{k}^{2}}{2}$). We can also find the distribution for ${\bar{X}}_{\Delta }$ unconditional on $\mu_{\Delta }$ as

(21) $${\bar{X}}_{\Delta }|\sigma_{0}^{2},y_{1},\cdots ,y_{K}\dot{∼}N\left(\sum p_{k}\lambda_{k},\left(\frac{1}{n_{A}}+\frac{1}{n_{B}}\right)\sigma_{0}^{2}+\sum_{k}p_{k}^{2}\xi_{k}^{2}\right);$$

see the derivation also in Section E of the Supporting Information. Apparently, this marginal distribution for ${\bar{X}}_{\Delta }$ relies on prior distribution for the unknown $\sigma_{0}^{2}$, which may yield different solutions of $n_{A}$ and $n_{B}$ across the Bayesian SSD criteria considered in this paper.

Let the interval $\left(a,a+\ell_{0}\right)$ be symmetric about $\mu_{N}$ given the marginal posterior for $\mu_{\Delta }$ in (19). The sample size is found requiring $P[|\mu_{\Delta }-\mu_{N}|\leq \frac{\ell_{0}}{2}|y_{1},\cdots ,y_{K},y_{K+1}]\geq 1-\alpha$, based on the ACC; thus

(22) $$\frac{\ell_{0}}{2\sigma_{N}}=\sqrt{\frac{1}{\sum p_{k}^{2}\xi_{k}^{2}}+\frac{1}{\left(\frac{1}{n_{A}}+\frac{1}{n_{B}}\right)\sigma_{0}^{2}}}\cdot \frac{\ell_{0}}{2}\geq z_{\alpha /2},$$

where $z_{\alpha /2}$ denotes the upper (α/2)‐th quantile of the standard normal distribution. We rewrite the expression and obtain

(23) $$\frac{n_{A}n_{B}}{n_{A}+n_{B}}\geq \left(\frac{4z_{\alpha /2}^{2}}{\ell_{0}^{2}}-\frac{1}{\sum p_{k}^{2}\xi_{k}^{2}}\right)\int_{0}^{\infty }\sigma_{0}^{2}g\left(\sigma_{0}^{2}\right)d\sigma_{0}^{2},$$

where $g(\sigma_{0}^{2})$ is the probability density function of an Inv‐Gamma($\frac{c}{2},\frac{c\sum p_{k}^{2}\xi_{k}^{2}}{2}$) distribution. The reader may compare this inequality with what was obtained for cases where $\sigma_{0}^{2}$ is known in (16).

Applying the ALC, we need to average the random credible interval length $\ell^{\prime }\left({\bar{x}}_{\Delta }\right)=2z_{\alpha_{0}/2}\sigma_{N}$ over the marginal distribution for ${\bar{X}}_{\Delta }$ which varies with $\sigma_{0}^{2}$. According to the definition of ALC, we obtain that

(24) $$\begin{array}{c} 2z_{\alpha_{0}/2}\int_{0}^{\infty }{\left(\frac{1}{\sum p_{k}^{2}\xi_{k}^{2}}+\frac{1}{\left(\frac{1}{n_{A}}+\frac{1}{n_{B}}\right)\sigma_{0}^{2}}\right)}^{-\frac{1}{2}}g\left(\sigma_{0}^{2}\right)d\sigma_{0}^{2}\leq \ell , \end{array}$$

which does not have a closed‐form solution. This requires a search over the integers for $n_{A}$ and $n_{B}$ to find the smallest sum that satisfies the inequality. With the APVC, we would likewise remove the dependence on $\sigma_{0}^{2}$ by integration. The formula thus becomes

(25) $$\frac{n_{A}n_{B}}{n_{A}+n_{B}}\geq \left(\frac{1}{\varepsilon_{0}}-\frac{1}{\sum p_{k}^{2}\xi_{k}^{2}}\right)\int_{0}^{\infty }\sigma_{0}^{2}g\left(\sigma_{0}^{2}\right)d\sigma_{0}^{2}.$$

## 3 APPLICATION

Hampson *et al.* (2014) present a Bayesian approach for elicitation of expert opinion on model parameters for enhanced design and analysis of rare‐disease trials. An elicitation meeting (Hampson *et al.*, 2015) was held for the MYPAN trial, which compares the efficacy of a new treatment (labeled *A*) relative to the standard of care (labeled *B*) for polyarteritis nodosa, a rare and severe inflammatory blood vessel disease. Priors were elicited from the input of 15 experts individually. Specifically, opinion was sought on (i) the probability that a patient given *B* would achieve disease remission within 6 months (a dichotomous event) and (ii) the log‐odds ratio of remission rates. Consensus distributions for the remission rates were obtained, with the mode at 71% for *A* and 74% for *B*.

In line with the original assumptions for the MYPAN trial, we suppose the Bernoulli probability is not close to 0 or 1, so the log‐odds ratio of treatment benefit, that is, $\theta_{k}=\log\left[\left(\rho_{Ak}\left(1-\rho_{Bk}\right)\right)/\left(\left(1-\rho_{Ak}\right)\rho_{Bk}\right)\right]$, would be approximately normally distributed (Agresti, 2003). Here, $\rho_{jk}$ denotes the probability of remission for patients receiving treatment j=A,B. We regard the expert opinion as a type of pretrial information and further assume it had been summarized in the form of $\theta_{k}|y_{k}∼N\left(m_{k},s_{k}^{2}\right),\;k=1,\cdots ,K$. Eliciting such expert opinion is a nontrivial problem; we refer the interested reader to the literature such as Dias *et al.* (2017). Furthermore, Hampson *et al.* (2014) detailed the elicitation process for reaching a probabilistic summary for the log‐odds ratio. For illustrative purposes, we assume five sets of expert opinion had been summarized as N(−0.26,0.25),N(−0.24,0.23), N(−0.37,0.22), N(−0.34,0.36), and N(−0.32,0.26) to inform $\theta_{k}$. The opinion would also be sought on $w_{k},\;k=1,\cdots ,5$ , to represent the experts' skepticism about the predictability of each pretrial parameter $\theta_{k}$ towards the parameter $\mu_{\Delta }$, measured on the continuous scale of 0 to 1. In this example, we suppose such pretrial information is valued about equally, with $w_{1}=0.15,\;w_{2}=0.20,\;w_{3}=0.17,\;w_{4}=0.13,\;w_{5}=0.20$. In practice, the trial statistician could look into the levels of pairwise commensurability between the $N(m_{k},s_{k}^{2})$ distributions through a discrepancy measure, such as the Hellinger distance (Dey and Birmiwal, 1994), to reconcile the choices of value for $w_{k}$.

For reaching a collective prior for $\mu_{\Delta }|y_{1},\cdots ,y_{5}$, synthesis weights $p_{1},\cdots ,p_{5}$ need to be specified. We apply a decreasing function:

(26) $$p_{k}=\frac{\exp(-w_{k}^{2}/s_{0})}{\sum_{k}\exp\left(-w_{k}^{2}/s_{0}\right)},$$

with a concentration parameter *s*_0_ to transform these weights from $w_{1},\cdots ,w_{K}$. Specifically, for $s_{0}\gg w_{k}$, all $p_{k}$ will be close to 1/K irrespective of the values of $w_{k}$. Whereas, with $s_{0}\to 0^{+}$, the smallest $w_{k}$ would have $p_{k}\to 1$, meaning that the corresponding $\theta_{k}|y_{k}$ tends to dominate the collective prior. The rationale behind this approach is that both $w_{k}$ and $p_{k}$ might be determined by some distance measure between parameters $\theta_{k}$ and $\mu_{\Delta }$. It is an objective‐directed approach, since we hope to discount preexperimental information to a larger extent via small values of $p_{k}$, when it is believed a priori to be less commensurate (thus, large values of $w_{k}$) with the new experimental data. Figures S2 and S3 (in the Supporting Information) visualize the impact of $w_{k},\;k=1,\cdots ,5$, and *s*_0_ on the informativeness of the collective prior. A thorough evaluation by Zheng and Wason (2022) shows this objective‐directed approach has desirable properties. We generally recommend choosing a small value (relative to the magnitudes of $w_{k}$) for *s*_0_, particularly because this can discern the degree of relevance and can further lead to a heavy‐tailed collective prior for cases of divergent pretrial information. Here, we set $s_{0}=0.05$ for illustration; consequently, $p_{1}=0.23,\;p_{2}=0.16,\;p_{3}=0.20,\;p_{4}=0.25,\;p_{5}=0.16$. This gives a collective prior $\mu_{\Delta }|y_{1},\cdots ,y_{5}∼N\left(-0.309,0.154\right)$, when specifying $\nu_{k}∼w_{k}\text{Gamma}\left(2,2\right)+\left(1-w_{k}\right)\text{Gamma}\left(18,3\right)$ for our model.

Assuming known variance of $\sigma_{0}^{2}=0.35$ and that $n_{A}=n_{B}$, the total sample sizes (i.e., $n_{A}+n_{B}$) found based on the ACC and ALC criteria are both 41.8 for 95% posterior coverage probability and the credible interval length as 0.65 on average. For cases of unknown $\sigma_{0}^{2}$, we let $\sigma_{0}^{2}∼$ Inv‐Gamma(2.500, 0.385) (i.e., setting c=5). The ACC and ALC sample sizes become 30.7 and 24 for attaining the same posterior behaviors, respectively. Targeting $\varepsilon_{0}=0.03$, the APVC sample sizes are 32.2 and 27.6 for known and unknown $\sigma_{0}^{2}$, respectively.

It may be counterintuitive to find the sample size for cases of unknown variance is smaller than those for known variance here, especially if the latter is perceived as a version of the former with infinite precision. We would reiterate that the prior specification for $\sigma_{0}^{2}$ in our methodology uses pretrial information, via an Inv‐Gamma($\frac{c}{2},\frac{c\sum p_{k}^{2}\xi_{k}^{2}}{2}$) distribution. Taking the mode for illustration, the sample size would be proportional to the quantity $\frac{c}{c+2}\sum p_{k}^{2}\xi_{k}^{2}$, that is, the magnitude of the collective prior variance (i.e., 0.154 in this illustration) scaled by the constant relying on *c*. This is smaller than the fixed $\sigma_{0}^{2}=0.35$; so not surprisingly, a smaller sample size would be yielded by the same criterion. We also caution that the distribution is not necessarily symmetric about the mode, and the uncertainty in $\sigma_{0}^{2}$ needs to be integrated out for the formal SSD.

## 4 PERFORMANCE EVALUATION

### 4.1 Basic settings

Motivated by the MYPAN trial, we generate four base scenarios of historical data, which are configured with different levels of pairwise (in)commensurability and informativeness. Such preexperimental information from *K* sources is supposed to have been summarized as $\theta_{k}|y_{k}∼N\left(m_{k},s_{k}^{2}\right),\;k=1,\cdots ,K$. For each base scenario, two distinct sets of prior mixture weights I and II for robust borrowing are considered to implement the proposed approach for borrowing of information, as listed in Table 1. These fractions are chosen to (a) reflect high and low levels of prior confidence in the historical data when they are consistent between themselves or (b) designate a certain source of historical data to be more influential.

**TABLE 1:** Configurations of hypothetical historical data, each accompanied by two sets of weights for robust borrowing of information. preexperimental information about $\theta_{k}|y_{k}$ is assumed to have been summarized by a $N(m_{k},s_{k}^{2})$ prior for k=1,⋯,5

			Hypothetical historical data						
			k=1	k=2	k=3	k=4	k=5	$\sum p_{k}\lambda_{k}$	$\sum p_{k}^{2}\xi_{k}^{2}$
Configuration 1		$m_{k}$	−0.260	−0.240	−0.370	−0.340	−0.320		
		$s_{k}^{2}$	0.250	0.230	0.220	0.360	0.260		
	Robust weights I	$w_{k}$	0.103	0.175	0.081	0.143	0.077	−0.311	0.129
		$p_{k}$	0.214	0.143	0.232	0.176	0.235		
	Robust weights II	$w_{k}$	0.252	0.319	0.140	0.306	0.149	−0.325	0.198
		$p_{k}$	0.149	0.069	0.359	0.082	0.341		
Configuration 2		$m_{k}$	−0.260	−0.240	−0.370	−0.340	−0.320		
		$s_{k}^{2}$	0.100	0.100	0.100	0.100	0.100		
	Robust weights I	$w_{k}$	0.103	0.175	0.081	0.143	0.077	−0.311	0.096
		$p_{k}$	0.214	0.143	0.232	0.176	0.235		
	Robust weights II	$w_{k}$	0.252	0.319	0.140	0.306	0.149	−0.325	0.158
		$p_{k}$	0.149	0.069	0.359	0.082	0.341		
Configuration 3		$m_{k}$	−0.260	−0.170	−0.440	−0.150	0.120		
		$s_{k}^{2}$	0.250	0.640	0.970	1.540	0.590		
	Robust weights I	$w_{k}$	0.101	0.219	0.385	0.385	0.304	−0.198	0.295
		$p_{k}$	0.559	0.263	0.035	0.035	0.108		
	Robust weights II	$w_{k}$	0.325	0.203	0.171	0.180	0.272	−0.215	0.379
		$p_{k}$	0.065	0.235	0.298	0.280	0.122		
Configuration 4		$m_{k}$	−0.260	−0.170	−0.440	−0.150	0.120		
		$s_{k}^{2}$	0.250	0.150	0.400	0.890	0.220		
	Robust weights I	$w_{k}$	0.066	0.303	0.459	0.355	0.115	−0.099	0.226
		$p_{k}$	0.473	0.082	0.008	0.041	0.396		
	Robust weights II	$w_{k}$	0.537	0.306	0.054	0.220	0.350	−0.312	0.343
		$p_{k}$	0.002	0.098	0.602	0.243	0.055		

We compute the Hellinger distances of any two $N(m_{k},s_{k}^{2})$ distributions to describe their pairwise (in)commensurability, as visualized in Figure S4 of the Supporting Information. This is used to justify the values of $w_{k}$ in Table 1 for our numerical study being no greater than 0.500, as the largest Hellinger distance in Figure S4 is below 0.500. Both the Gamma mixture prior for $\nu_{k}$, and derivation of the weights, $p_{k}$, for prioritizing certain historical data to form a collective prior, follow our specification in Section 3. Nonetheless, we note at the outset the Gamma component distributions can be equally essential, as choices have an impact on the effective sample size of the collective prior (Neuenschwander *et al.*, 2020).

We compare the sample sizes computed using the proposed Bayesian SSD formulae with those computed (a) without robustification, that is, setting each $w_{k}=0$ for k=1,⋯,5, (b) without leveraging historical information for $\mu_{\Delta }$, that is, setting each $w_{k}=1$, (c) from the proper Bayesian SSD approach driven by a single prior, here specified as the most informative $N(m_{k},s_{k}^{2})$, for example, N(−0.37,0.22) for configuration 1, and (d) from an optimal approach as the benchmark. Specifically, the optimal approach is coupled with a perfectly commensurate prior, by equating $\sigma_{0}^{2}$ to the collective prior variance $\sum p_{k}^{2}\xi_{k}^{2}$. In this way, the corresponding result would serve as the benchmark referring to the scenario of perfect consistency between the collective prior and the new data, so the largest saving in sample size could be attained by the proposed methodology. For cases of unknown $\sigma_{0}^{2}$, the optimal sample sizes could be approached by setting *c* to a sufficiently large value.

### 4.2 Results

**FIGURE 1:**
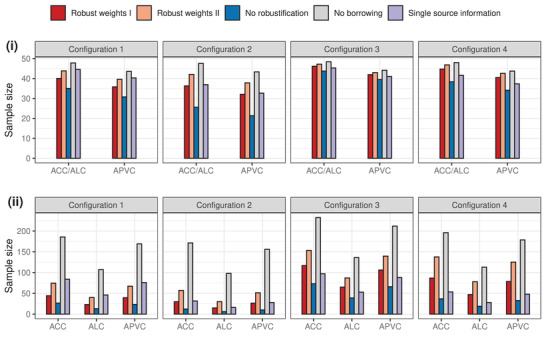
Comparison of the Bayesian SSD approaches in terms of the sample size obtained according to the ACC, ALC, and APVC criteria for cases of (i) known $\sigma_{0}^{2}=0.35$ and (ii) unknown $\sigma_{0}^{2}$. Sample sizes in subfigure (ii) for unknown $\sigma_{0}^{2}$ are computed setting c=3, that is, assuming that $\sigma_{0}^{2}∼$ Inv‐Gamma$\left(1.5,1.5\times \sum p_{k}^{2}\xi_{k}^{2}\right)$, for fairly limited use of preexperimental information to inform the variance $\sigma_{0}^{2}$. This figure appears in color in the electronic version of this article, and any mention of color refers to that version

Figure 1 visualizes a subset of the results, which compare the proposed Bayesian SSD formulae using robust weights I and II with the alternative approaches for cases of known and unknown $\sigma_{0}^{2}$, respectively. Here, we assume $\sigma_{0}^{2}=0.35$ and, if unknown, $\sigma_{0}^{2}∼$ Inv‐Gamma(1.5, 1.5$\times \sum p_{k}^{2}\xi_{k}^{2}$) for illustration. We fix the posterior credible interval length $\ell_{0}=0.65$ to find the ACC sample sizes, so that the average coverage probability would be 95%, that is, targeting α=0.05 in (16). Likewise, for computing the ALC sample sizes, we fix $\alpha_{0}=0.05$ and constrain the average length of the posterior credible interval below 0.65. When applying the APVC, sample sizes are found with the average posterior variance retained to level ε=0.03.

In all configurations 1–4, we see that the sample sizes computed according to the same criterion, using robust weights I, are smaller than those using robust weights II. This is because following our setting the collective prior, produced by robust weights I, has a smaller variance than its counterpart by robust weights II, for each configuration. Moreover, sample sizes yielded using either robust weights I or II are always bounded by those using no robustification ($w_{k}=0$) and no borrowing ($w_{k}=1$). We may think that no robustification leads to the least conservative result by the proposed SSD formulae, for the given historical information fully used. These, however, are not necessarily identical to the optimal situations, where $\sigma_{0}^{2}$ is equated to the collective prior variance, or largely determined by the latter if unknown. In Figure 1, we omit the benchmark optimal sample sizes that may be obtained by using the proposed formulae with robust weights I and II for each configuration. Yet we will comment on the maximal saving that the proposed SSD approach can achieve in the following along with other figures.

The height difference across bars of sample sizes, computed using our approach with robust weights I or II and no borrowing ($w_{k}=1$), quantifies the benefit from leveraging preexperimental information for $\mu_{\Delta }$. Looking across subfigures (i) and (ii), such height differences between methods are far greater for the unknown variance case than the known variance case. Comparison of SSD approaches with borrowing versus no borrowing, as visualized in subfigure (ii) of Figure 1, would be more objective for illustrating the benefit. As mentioned, choosing c=3 means $\sigma_{0}^{2}$ would be related with $\sum p_{k}^{2}\xi_{k}^{2}$ to a very limited extent, as if a diffuse prior had been placed on $\sigma_{0}^{2}$. Thereby, implementing no borrowing by setting $w_{k}=1$, preexperimental information would neither be leveraged through the robust prior for $\mu_{\Delta }$, nor through the prior for the unknown $\sigma_{0}^{2}∼$ Inv‐Gamma($\frac{c}{2},\frac{c\sum p_{k}^{2}\xi_{k}^{2}}{2}$). Consequently, larger sample sizes would be found for no borrowing SSD for the unknown $\sigma_{0}^{2}$ than the known cases assuming $\sigma_{0}^{2}=0.35$, to retain similar properties of the posterior distribution. Focusing on the bars for robust weights I and II against no borrowing within subfigure (ii), saving in all the ACC, ALC, and APVC sample sizes could be as much as two‐thirds for configurations 1 and 2. Such saving is attenuated in configurations 3 and 4 when historical information is divergent. In configurations 3, the ACC (ALC) sample size obtained from the no borrowing approach is about twice the size from the proposed approach with robust weights I, specifically, 232.2 versus 116.8 (136 vs. 65), respectively. We observe a small increase in sample size by using robust weights II instead of I, because slightly higher prior probabilities of incommensurability had been allocated to certain informative $N(m_{k},s_{k}^{2})$ for greater down‐weighting. The trend is similar for results in configuration 4.

We then compare the proposed approach with an alternative strategy, that is, restricting the use of preexperimental information from a single source. When the historical data are consistent (divergent) between themselves, the proposed SSD formulae lead to smaller (larger) sample sizes, as presented obviously in configuration 1 (configurations 3 and 4) for both cases of known and unknown $\sigma_{0}^{2}$. As one may perceive, such selection of a single source could be less robust than averaging over all available preexperimental information. Another noteworthy finding is concerned with the comparison of the ACC and ALC sample sizes, particularly when $\sigma_{0}^{2}$ is unknown and we place a minimally informative prior on it (setting c=3). As shown in Figure 1, the ALC sample size is universally smaller than the ACC sample size for all these investigated configurations.

**FIGURE 2:**
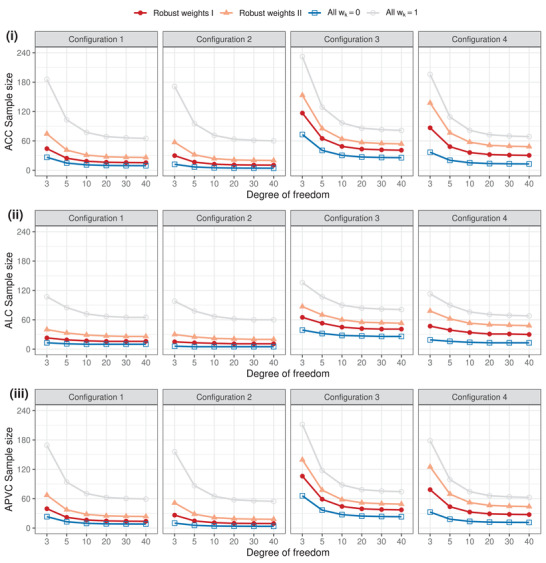
The ACC, ALC, and APVC sample sizes for the new trial, where the unknown $\sigma_{0}^{2}$ could be related to the collective prior variance by assuming the quantity $c\sum p_{k}^{2}\xi_{k}^{2}/\sigma_{0}^{2}∼\chi^{2}\left(c\right)$. The extent of borrowing for better knowledge about $\sigma_{0}^{2}$ depends on the number of degrees of freedom, *c*. This figure appears in color in the electronic version of this article, and any mention of color refers to that version

We move on to quantify how the sample sizes would vary as *c* changes. Focusing on approaches using preexperimental information from multiple sources, Figure 2 displays the sample sizes exclusively for cases of unknown $\sigma_{0}^{2}∼$ Inv‐Gamma($\frac{c}{2},\frac{c\sum p_{k}^{2}\xi_{k}^{2}}{2}$). We set c=3,5,10,20,30,40, and keep the target level of each SSD criterion unchanged from what we have used for Figure 1. As *c* gets larger, the sample sizes for all approaches investigated here decrease and tend to stabilize at their own lowest levels possible. This could be explained from the perspective of prior effective sample size, to which variance is a key determining factor. Consider the prior placed on the inverse of the unknown variance that $\frac{1}{\sigma_{0}^{2}}∼$ Gamma($\frac{c}{2},\frac{c\sum p_{k}^{2}\xi_{k}^{2}}{2}$), of which the mean and variance are $\frac{1}{\sum p_{k}^{2}\xi_{k}^{2}}$ and $\frac{2}{c}\cdot \frac{1}{{\left(\sum p_{k}^{2}\xi_{k}^{2}\right)}^{2}}$, respectively. As *c* increases, the prior variance diminishes, meaning that possible values of $\frac{1}{\sigma_{0}^{2}}$ are more concentrated around the prior mean obtained based on historical data. For c≥20, the ACC and ALC sample sizes are nearly identical. Whereas, the ACC sample size is more sensitive than the ALC to small values of *c*, for example, when c=3,5. We note that the so‐called “no borrowing” (by setting $w_{k}=1$) should be clarified as no borrowing in terms of the parameter $\mu_{\Delta }$. When *c* gets larger, it means the unknown variance $\sigma_{0}^{2}$ would be more closely tied to the prior variance based on the historical data. That is, borrowing is enabled through the variance, although not directly the parameter of inferential interest. By fixing $w_{k}=1$, historical data would not be leveraged through the robust prior for $\mu_{\Delta }$, but nevertheless could be used to inform the unknown $\sigma_{0}^{2}$, particularly when *c* is sufficiently large.

**FIGURE 3:**
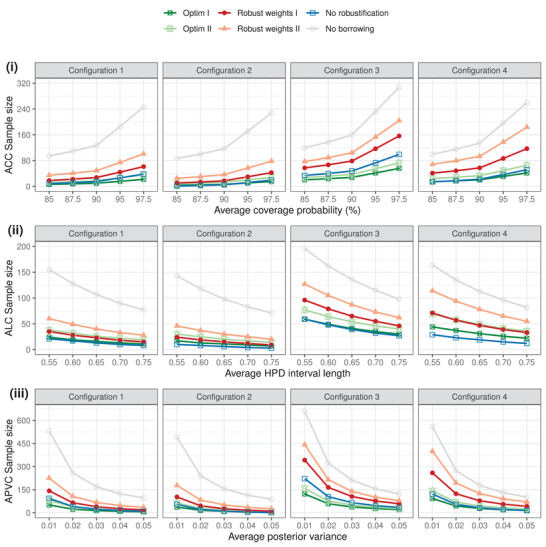
Sample sizes required when $\sigma_{0}^{2}$ is unknown to retain the desired average property of the posterior distribution. The ACC and ALC sample sizes are computed by fixing the credible interval length $\ell_{0}=0.65$ and coverage probability $1-\alpha_{0}=95$%, respectively. This figure appears in color in the electronic version of this article, and any mention of color refers to that version

Figure 3 illustrates how the sample size varies, for cases of unknown $\sigma_{0}^{2}$, when targeting the average coverage probability, posterior credible length, and posterior variance at different levels. Like in Figure 1, these results are obtained by setting c=3 for the very limited use of preexperimental information to inform $\sigma_{0}^{2}$. The optimal sample sizes are also plotted to show the maximal saving the proposed SSD formulae may achieve. Specifically, Optim I and II should be taken as the benchmark for formulae using robust weights I and II, respectively. As expected, sample sizes by robust weights I and II would always be bounded by the extremes of no robustification (all $w_{k}=0$) and no borrowing (all $w_{k}=1$). Given a fixed length $\ell_{0}=0.65$ of the HPD interval, more ACC sample sizes would be required if increasing the desired coverage probability on average, 1−α. For example, the ACC sample size computed using robust weights I (II) rises from 78.7 to 156.5 (104.4 to 204.2) for configuration 3 had the level of 1−α been lifted from 90% to 97.5%. The displayed ALC sample sizes in subfigure (ii) ensure the coverage probability as 95%; by relaxing the target average HPD interval length, fewer sample sizes would be needed. Likewise, the APVC sample sizes in subfigure (iii) share this commonality of decreasing as we relax the target posterior variance. Generating these plots would be helpful in practice for balancing between obtaining an economic sample size planning and a posterior sufficiently informative for inferences on a case‐by‐case basis. For example, targeting the average length of the HPD interval with 95% coverage probability as ℓ=0.60 requires the ALC sample size to be 28 for configuration 1 using robust weights I, which may not be much different from 23 yielded by the level ℓ=0.65.

We further investigate the impact of $s_{k}^{2}$, the associated levels of uncertainty inherent to historical data k=1,⋯,K, on the respective sample sizes. Configurations 1 and 2, with the robust weights kept the same, have been constructed for this purpose. From Figures 1, 2, 3, it is clear that Configuration 1 requires a larger sample size than Configuration 2 under the same criterion. The explanation is that Configuration 2, with smaller sample variation, leads to a more informative collective prior for $\mu_{\Delta }$, so less information (sample size) would be required from the new experiment for the inference.

**FIGURE 4:**
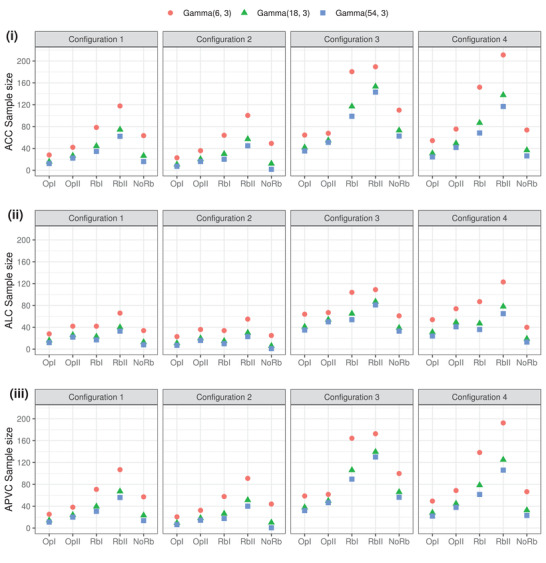
The proposed Bayesian SSD is dependent on the choice of the informative Gamma component distribution for strong borrowing. The labels at the *x*‐axis are short for Optim I, Optim II, Robust weights I, Robust weights II, and no robustification, respectively. This figure appears in color in the electronic version of this article, and any mention of color refers to that version

We also examine how sensitive the proposed Bayesian SSD formulae are to the Gamma mixture components. Since a suitable yet least informative Gamma$\left(a_{01},\;b_{01}\right)$ has been chosen for down‐weighting, the other component of the mixture prior, Gamma$\left(a_{02},\;b_{02}\right)$, determines the maximum borrowing possible. Assuming unknown $\sigma_{0}^{2}$ and setting c=3, Figure 4 shows the Bayesian SSD under different choices of the hyperparameters, *a*_02_ and *b*_02_, for each criterion. As expected, a more informative Gamma$\left(a_{02},\;b_{02}\right)$ yields a smaller sample size given the same set of $w_{k},\;k=1,\cdots ,K$. The ALC sample sizes appear to have least decreasing, compared with the ACC and APVC, in this sensitivity evaluation. We also observe that the reduction in Bayesian sample sizes is not proportional to the improving of informativeness of Gamma$\left(a_{02},\;b_{02}\right)$: setting the informative component as Gamma(18, 3) is not much different from Gamma(54, 3) for our illustrative examples. For practical implementation, we recommend the component Gamma distributions to be chosen for representing two extremes of very limited borrowing and complete pooling of information, when given a full prior mixture weight $w_{k}=1$ and $w_{k}=0$, respectively.

Finally, comprehensive simulation studies have been performed in Section H–J of the Supporting Information to investigate (i) the average properties of the posterior for $\mu_{\Delta }$ as updated by the new experimental data, (ii) the sensitivity to nonnormal data, and (iii) the performance if original priors (without normal approximation) are used for the analysis.

## 5 DISCUSSION

Planning a new experiment with a sufficient sample size necessitates the use of relevant information. Bayesian methods allow for the inherent uncertainty in the estimate of model parameters, as well as a formal incorporation of any expert opinion or historical data. In this paper, we have developed Bayesian sample size formulae that use commensurate priors to leverage preexperimental data, available from multiple sources, for the model parameter(s) of interest. While we note proposals based on the “two‐prior” approach (De Santis, 2007; Brutti *et al.*, 2009), the proposed method specifies a singular prior for both the design and analysis of the new experiment.

One area that deserves more investigation is surrounding $w_{k}$. Following Zheng and Wason (2022), we recommend these are based on some measures of distributional discrepancy, such as the Hellinger distance between any two $N(m_{k},s_{k}^{2})$ distributions. The underlying logic is that the new experiment, at the planning stage, is regarded as compatible with the historical experiments, then their data would also be. The levels of the (in)commensurability between a preexperimental parameter and the new experimental parameter would thus be comparable to those between the preexperimental parameters themselves. Nevertheless, we recognize that these prior mixture weights $w_{k}$ cannot be correctly specified when the new experimental data are yet to be generated. Pragmatically, the new experiment could be embedded with interim analyses to enable midcourse modifications towards $w_{k}$. Each update in terms of $w_{k}$ tends to better reflect the genuine incommensurability (Zheng and Hampson, 2020).

As noted by one reviewer, there are circumstances that preexperimental information may be available for a single arm (say, to inform $\mu_{A}$ or $\mu_{B}$) only. The proposed Bayesian methodology can still be useful in that the information may be represented into a commensurate predictive prior to the arm‐based statistic(s). Analytical derivation of a posterior for the mean difference can follow our one presented in Section 2. This would be particularly relevant to the special topic of using historical control in clinical trials to supplement or replace a concurrent control. However, we caution that the selection of relevant pretrial data on one arm needs to be done carefully, since the model may introduce systematic difference between arms that would affect the inference of the difference in means.

For comparing two Bernoulli probabilities in Section 3, we used a logit transformation to consider the log‐odds ratio, which is generally adequately modeled by a normal distribution. The approach of constructing a normal statistic can also be used for time‐to‐event data, which is elaborated upon in Section K of the Supporting Information with new formulae presented. We are aware of the limitations. For example, accurate estimation of the Bernoulli probabilities is not straightforward and the censoring assumptions in the time‐to‐event data are simplified. We hope this work motivates further research for SSD in both binomial and time‐to‐event data within this Bayesian context.

Throughout this paper, we supposed preexperimental information had been available with regard to the parameter of influential interest. Situations may be more complex in practice. For instance, historical data may have been recorded on a different measurement scale (Zheng *et al.*, 2020) from what might be for the new experiment under planning. This is an area where our future research would look towards. To promote the uptake of our methodology, we have summarized the necessary actions, along with the specification of key parameters, at different stages of the planning of a new experiment in Section L of the Supporting Information. As a separate note, we applied quite general criteria such as ACC and ALC to control the average coverage probability or length of the HPD interval of the posterior distribution for the parameter of influential interest throughout. In such decision frameworks, the sample size largely depends on the informativeness of a prior distribution for $\mu_{\Delta }$, as well as for $\sigma_{0}^{2}$ when using preexperimental data to inform the variance. With each criterion concerning an average property of the posterior distribution, permitting borrowing (with $0<w_{k}<1$) yields a smaller sample size than the approach of no borrowing (which can be a limiting case of the proposed model with $w_{k}=1$). However, when alternative decision criteria are applied, it is not necessarily true that enabling borrowing always leads to a sample size reduction. An example is research for overcoming prior‐data conflict, where the prior mismatches the data accrued from the new experiment. There is relevant literature addressing the issue in clinical trials, where maintaining strong control of error rates is required by regulatory agencies (EMA, 1998). Our sample size formulae according to the ACC can be closely relevant for giving a solution analogous to the frequentist hypothesis testing; for example, rejection of the null hypothesis could be defined based on posterior interval probabilities with respect to a magnitude of effect deemed clinically meaningful (Whitehead *et al.*, 2008).

### OPEN RESEARCH BADGES

This article has earned Open Data and Open Materials badges. Data and code are available at https://github.com/haiyanzheng/SSDcmspriors.

## Supporting information

Web Appendices A–E referenced in Section 2, Figures S2– S4 in Sections 3 and 4, and Appendices H–L for additional simulations, extended application to time‐to‐event data, and a brief user‐guide to apply the proposed methodology, are available at the Biometrics website on Wiley Online Library. Programming code for the sample size formulae and reproducing the numerical results, is posted online along with this paper, as well as available at GitHub: https://github.com/haiyanzheng/SSDcmspriors

## Data Availability

The authors confirm that the simulated data supporting the findings of this paper are reproducible with openly available R code in the Supporting Information.
